# The Table Turning Delusion

**Published:** 1853-10

**Authors:** 


*The Table Turning Delusion.*—The following letter from Professor Faraday is extracted from the *Times* of Thursday last:

*To the Editor of the Times:*—Sir: I have recently been engaged in the investigation of table-turning. I should be sorry that you should suppose I thought this necessary on my own account, for my conclusion respecting its nature was soon arrived at, and is not changed; but I have been so often misquoted, and applications to me for an opinion are so numerous, that I hoped, if I enabled myself by experiment to give a strong one, you would consent to convey it to all persons interested in the matter. The effect produced by table-turners has been referred to electricity, to magnetism, to attraction, to some unknown or hitherto unrecognized physical power able to affect inanimate bodies—to the revolution of the earth, and even to diabolical or supernatural agency. The natural philosopher can investigate all these supposed causes but the last; that must to him, be too much connected with credulity or superstition to require any attention on his part.

Believing that the first cause assigned—namely, a *quasi* involuntary muscular action (for the effect is with many subject to the wish or will)—was the true cause, the first point was to prevent the mind of the turner having an undue influence over the effects produced in relation to the nature of the substances employed. A bundle of plates consisting of sand-paper, millboard, glue, glass, plastic clay, tinfoil, card-board, gutta percha, vulcanized caoutchouc, wood, and resinous cement, was therefore made up and tied together, and being placed on a table under the hand of a turner, did not prevent the transmission of the power; the table turned or moved exactly as if the bundle had been away, to the full satisfaction of all present. The experiment was repeated, with various substances and persons, and at various times, with constant success; and henceforth no objection could be taken to the use of these substances in the construction of apparatus. The next point was to determine the place and source of motion—*i. e.*, whether the table moved the hand or the hand moved the table; and for this purpose indicators were constructed. One of these consisted of a light lever, having its fulcrum on the table, its short arm attached to a pin fixed on a card-board, which could slip on the surface of the table, and its long arm projecting as an index of motion. It is evident that if the experimenter willed the table to move toward the left, and it did so move *before* the hands, placed at the time on the card-board, then the index would move to the left also, the fulcrum going with the table. If the hands involuntarily moved toward the left *without* the table, the index would go toward the right; and if neither table nor hands moved, the index would itself remain immovable. The result was that when the parties saw the index it remained very steady; when it was hidden from them, or they looked away from it, it wavered about though they believed that they always pressed directly downward; and when the table did not move, there was still a resultant of hand force in the direction in which it was wished the table should move, which, however, was exercised quite unwittingly by the party operating. This resultant it is which, in the course of the waiting time, while the fingers and hands become stiff, numb and insensible by continued pressure, grows up to an amount sufficient to move the table or the substances pressed upon. But the most valuable effect of this test-apparatus (which was afterward made more perfect and independent of the table) is the corrective power it possesses over the mind of the table-turner. As soon as the index is placed before the most earnest, and they perceive—as in my presence they have always done—that it tells truly whether they are pressing downward only or obliquely, then all effects of table-turning cease, even though the parties persevere, earnestly desiring motion, till they become weary and worn out. No prompting or checking of the hands is needed—*the power is gone;* and this only because the parties are made conscious of what they are really doing mechanically, and so are enabled unwittingly to deceive themselves. I know that some may say that it is the card-board next the fingers which moves first, and that *it* both drags the table and also the table-turner with it. All I have to reply is, that the card-board may in practice be reduced to a thin sheet of paper weighing only a few grains, or to a piece of goldbeaters' skin, or even the end of the lever, and (in principle) to the very cuticle of the fingers itself. Then the results that follow are too absurd to be admitted: the table becomes an incumbrance, and a person holding out the fingers in the air, either naked or tipped with goldbeaters' skin or card-board, ought to be drawn about the room, &c.; but I refrain from considering imaginary yet consequent results which have nothing philosophical or real in them. I have been happy thus far in meeting with the most honorable and candid though most sanguine persons, and I believe the mental check which I propose will be available in the hands of all who desire truly to investigate the philosophy of the subject, and, being content to resign expectation, wish only to be led by the facts and the truth of nature. As I am unable, even at present, to answer all the letters that come to me regarding this matter, perhaps you will allow me to prevent any increase by saying that my apparatus may be seen at the shop of the philosophical instrument maker—Newman, 122 Regent street.

Permit me to say, before concluding, that I have been greatly startled by the revelation which this purely physical subject has made of the condition of the public mind. No doubt there are many persons who have formed a right judgment or used a cautious reserve, for I know several such, and public communications have shown it to be so; but their number is almost as nothing to the great body who have believed and borne testimony, as I think, in the cause of error. I do not here refer to the distinction of those who agree with me and those who differ. By the great body, I mean such as reject all consideration of the equality of cause and effect; who refer the results to electricity and magnetism—yet know nothing of the laws of these forces; or to attraction—yet show no phenomena of pure attractive power; or to the rotation of the earth, as if the earth revolved round the leg of a table; or to some unrecognized physical force, without inquiring whether the known forces are not sufficient; or who even refer them to diabolical or supernatural agency, rather than suspend their judgment, or acknowledge to themselves that they are not learned enough in these matters to decide on the nature of the action. I think the system of education that could leave the mental condition of the public body in the state in which this subject has found it must have been greatly deficient in some very important principle.

I am, sir, your very obedient servant,

M. Faraday.

Royal Institution, June 28, 1853.—*London Lancet.*

